# Congenital Displacement of the Hips

**Published:** 1892-02-13

**Authors:** 


### THE PRACTITIONER'S MIRROR AND RETROSPECT.

[*The Editor will be glad to receive offers of co-operation and contributions from members of the profession. All letters should be addressed to* THE EDITOR, THE LODGE, PORCHESTER SQUARE, LONDON, W.]

### SURGERY.

### CONGENITAL DISPLACEMENT OF THE HIPS.

Congenital displacement of the hips is not a very rare affection. Sometimes it is not recognised, and the secondary lateral curvature of the spine, from the shortening of the limb, is treated as if it were the primary disease.

The deformity is not observed before the child begins to walk, and it is doubtful whether the head is really displaced before this period, but when the child has begun to walk, the head gets forced up on the dorsum ilii, and a very characteristic waddling gait will be observed if the displacement is double, and on closer examination of the child lordosis will be apparent, and the raised prominent trochanters will be seen, and often the head felt rotating on the dorsum. If the affection is unilateral, besides a slight limp there may be few signs of its existence, as the tilting of the pelvis often compensates for the shortening. But what we do observe in these unilateral cases is lateral curvature of the spine, secondary to the true shortening of the limb; and we have seen a case of lateral curvature caused in this way treated by a spinal support, while the real cause of the curvature had been completely overlooked. A high sole to the boot is what is wanted in these cases to cure the lateral curvature of the spine. In all cases of lateral curvature of the spine in children, we should always make sure that it is not caused by shortening of either lower limb.

But there is no doubt that bilateral cases are not always recognised. The most marked case we have ever seen of double displacement was sent into hospital for "rheumatic contraction of the tendons," with a view to their division. The limbs could not be extended, and there was extreme lordosis; the muscles and tendons on the flexor aspect of the thigh certainly were contracted, but the primary lesion of congenital "dislocation" was evidenced by the raised prominent trochanters.

Recent investigation into the pathology of this disease has saved the accoucheur from blame. We know now that there is no normal acetabulum in which the head of the bone could lie. At a meeting of the Pathological Society in April, 1887, all the specimens which could be found in the London museums were exhibited, and a careful examination of them conclusively showed that the portion of the acetabulum which is formed by the ilium is wanting, and so a very imperfect acetabulum is formed, which could not contain the head. The head becomes more and more forced up on the dorsum. The ball and socket action is lost, and the gluteal and other external rotators degenerate, so that abduction and rotation outwards are not possible, or very limited. It is not necessary to go the round of the London museum to prove this. All the specimens are represented in a beautiful series of plates in the "Transactions of the Pathological Society" for 1887. These pictures show not only the malformation of the acetabulum and the displacement on the dorsum, but also a condition of the capsule which we shall presently see is of great importance. The head never leaves the capsule, there is no traumatism, but the head is displaced in the capsule, and thus we find an elongated loose capsule, which usually allows of a considerable amount of movement of the head. The ligamentum teres is often absent; when present it is elongated. Often the head is much flattened, and this is why sometimes it is so difficult to feel it rotating on the dorsum. A new acetabulum does not form, *i.e.*, no new bone is thrown out around the head, as happens sometimes in traumatic dislocation, because the head and dorsum ilii never come actually into contact, but are always separated by the capsular ligament, within which the head lies. Only a shallow depression is formed on the dorsum where the head has been rotating in the capsule.

Congenital dislocation of the hip is more common in girls than boys, and is hereditary. Adams found that in 20 out of 40 cases investigated there was no difficulty at birth. These facts, and the complete absence of any signs of old traumatism in all the cases examined, are additional arguments against the responsibility of the accoucheur in the production of the deformity.

And now we come to the very important question, what can be done for these cases? What they suffer from is the shortening of the limb in the unilateral displacement, and, in the bilateral, the tilting of the pelvis into an abnormally horizontal position. There is remarkably little flexion, adduction, or rotation inwards, such as we should get in a traumatic dislocation on the dorsum. In five cases of which we have notes, in only one (a bilateral case) was there any degree of flexion or adduction, but in this case the flexion was extreme in both limbs, and the adduction very marked in one. This is the only kind of case in which division of the muscles could do any good, but the muscles of flexion are not accessible to division, though the adductor longus is.

Clearly then, our only chance of doing good lies, as Adams has shown, in getting the head fixed near the acetabulum. Can we do this? Dr. Buckminster Brown, of Boston, treated a little girl with bilateral displacement by means of rest and extension carried out for thirteen months, and not only got the head in good position, but into a position in which it remained for two and a quarter years after treatment was discontinued. There was complete restoration of the natural form of the hips, and the lordosis was also completely removed. This case was published in 1885, and it would be very interesting to learn her condition now. It is usually quite easy to draw the head down into better position, but even if we can keep it there for a long time, what is to fix it in the better position? Adams thinks that the upper part of the capsule into which the head used to ascend before it was fixed by the treatment may become contracted, as the lower part certainly has in some museum specimens where the head occupies the upper part. He also thinks that the "adapted growth of the muscular and fibrous structures" will tend to keep the head in position. But surely any shortening of the muscles would tend rather to draw the head further upon the dorsum; and it does not seem to us that the treatment by prolonged rest and extension offers any reasonable prospect of the head becoming permanently fixed in the new position. It will, of course, retard the displacement upwards for some considerable time, but to keep a young child lying down for two years is a method of treatment not to be lightly undertaken, and this is the time prescribed by Adams, with another year's extension by means of apparatus when the child begins to get about again. In the two cases which he has already treated by two years' recumbency and extension, the shortening had almost completely gone at the end of that time, but when reported they were still using extension apparatus, and less than a year had elapsed since the recumbency was given up, and so we cannot yet judge of the permanent result. But, however difficult it may be to conceive how the head can get fixed in a better position, here, at least, is demonstration that not merely is the shortening arrested but actually diminished, from restoration of the head to a more normal resting place.

Then, again, Adams recommends that if continuous recumbency with extension cannot be carried out, half-way measures should be adopted. In cases of unilateral displacement, extension should be put on during recumbency, and the child get about with a high heel on the other leg, and crutches, and a pelvic band to steady the trochanters, with, if possible, the addition of an extension apparatus—like the American hip-joint instrument—on the affected limb. In bilateral affections he still recommends extension and recumbency as far as possible, but we do not see that much is to be hoped from this when the child is walking about at intervals.

But looking at the fact that unilateral cases get on fairly well with a high heel on the affected side, we should hardly be inclined to adopt any very tedious method of treatment, especially as it is yet to be shown how permanent the results will be; and even a markedly waddling gait from the bilateral displacement may not be worse than two year's restriction from walking at all, even in early childhood.

